# Improving the Processability and Performance of Micronized Fiber-Reinforced Green Composites through the Use of Biobased Additives

**DOI:** 10.3390/polym14173451

**Published:** 2022-08-24

**Authors:** Bruno F. A. Valente, Armando J. D. Silvestre, Carlos Pascoal Neto, Carla Vilela, Carmen S. R. Freire

**Affiliations:** 1 CICECO–Aveiro Institute of Materials, Department of Chemistry, University of Aveiro, 3810-193 Aveiro, Portugal; 2 RAIZ, Research Institute of Forest and Paper (The Navigator Company), Rua José Estevão, Eixo, 3800-783 Aveiro, Portugal

**Keywords:** green composites, poly(lactic acid), poly(hydroxybutyrate), cellulose, micronized fibers, epoxidized linseed oil, sugar-based surfactant, mechanical properties, thermal properties, biodegradability

## Abstract

Green composites made of bioplastics reinforced with natural fibers have gained considerable attention over recent years. However, the use of natural fibers in composites usually compromise some key properties, such as the impact strength and the processability of the final materials. In the present study, two distinct additives, namely an epoxidized linseed oil (ELO) and a sugar-based surfactant, viz. GlucoPure^®^ Sense (GPS), were tested in composite formulations of poly(lactic acid) (PLA) or poly(hydroxybutyrate) (PHB) reinforced with micronized pulp fibers. Both additives showed a plasticizing effect, which led to a decrease in the Young’s and flexural moduli and strengths. At the same time, the elongation and flexural strain at break were considerably improved on some formulations. The melt flow rate was also remarkably improved with the incorporation of the additives. In the PHB-based composites, an increment of 230% was observed upon incorporation of 7.5 wt.% ELO and, in composites based on PLA, an increase of around 155% was achieved with the introduction of 2.5 wt.% GPS. ELO also increased the impact strength to a maximum of 29 kJ m^−2^, in formulations with PLA. For most composites, a faster degradation rate was observed on the formulations with the additives, reaching, in the case of PHB composites with GPS, a noteworthy weight loss over 75% under burial testing in compost medium at room temperature.

## 1. Introduction

The global polymeric composites market is still dominated, in terms of value and volume, by composites based on petroleum-based matrices reinforced with glass fibers [1]. Nonetheless, natural fibers are becoming increasingly used as substitutes for glass fibers in composite materials due to their high availability, lower production cost, renewability and biodegradability [2]. Additionally, the manufacturing and processing of the composites is less hazardous when using natural fibers and causes less wear and tear on the machinery [2,3,4]. With a compound annual growth rate (CAGR) of 14.2% between 2020 and 2028, the natural fibers reinforced composites market is forecasted to reach over USD 60 billion, which clearly validates the establishment of natural fibers in the composites industry [5]. However, the sustainability of these so-called biocomposites is still not satisfactory, as the most common thermoplastic matrices, such as polypropylene (PP) or polyethylene (PE), are derived from non-renewable resources [6]. Additionally, the separation of the individual components of the composite is difficult, which makes them unsuitable for recycling, and disposal on landfills or incineration procedures are not sustainable options due to their negative long term impact on the environment [7,8,9].

To fabricate fully sustainable and environmentally friendly composites, all the components must have an entirely green path from the origin to its disposal. Poly(lactic acid) (PLA) and poly(hydroxybutyrate) (PHB) are good candidates to replace the conventional thermoplastic matrices given their biobased origins and biodegradability [10,11]. PLA, which can be produced by polycondensation of lactic acid or ring opening polymerization of a lactide intermediate, is a linear polyester with good mechanical properties, nontoxicity, stability to ultraviolet light and easily dyeable [12]. PHB is a polyester that is bio-synthetized by a wide variety of microorganisms (e.g., *Ralstonia eutropha*, *Aeromonas hydrophila*), belonging to the poly(hydroxyalkanoate) family, and with properties comparable to those of PP [11]. Despite their relatively high costs, both polymers are among the few bioplastics produced at a commercial level and can be easily processed through the mainstream technologies, such as injection molding, blow molding, thermoforming, and extrusion, among others, which facilitates their industrial processing and usage [11,12]. However, and although both polymers are promising for use as matrices in composites, their brittleness and low impact resistance still constitute a challenge [13,14].

Additionally, the combination of thermoplastic polymers (regardless of their biobased or fossil nature) with natural fibers, even though environmentally advantageous, still has some limitations. For instance, the hydrophilicity and high aspect ratio of the fibers makes them poorly compatible with the hydrophobic thermoplastic matrices and causes the formation of bundles or aggregates of fibers [15,16]. Likewise, the low impact strength, high water uptake and dimensional instability are also serious concerns for their long term application [6]. Mechanical treatments, such as milling processes or micronization, have been proposed to overcome the agglomeration issue by improving the dispersion of the fibers on the matrices through the decrease in the fiber size to the micrometer range [16,17,18]. Chemical pre-treatments of the fibers, i.e., alkali treatments [6] and surface modifications, such as acetylation, benzoylation or silylation, generally improve the compatibility between the composites constituents due to the hydrophobization of the fibers surface [6]. Additives, such as coupling agents, plasticizers, impact modifiers and lubricants, among others, may also be used to tailor the properties of composites, by improving the processability and minimizing some of the drawbacks mentioned before [13,19]. From an industrial perspective, the use of additives still provides an additional advantage as they are simpler to use and do not require multiple steps and time consuming processes, as in the case of the chemical modifications of the cellulose fibers [13].

Plasticizers are one of the most common class of additives used in thermoplastic and composite industries, being primarily applied to decrease the rigidity and brittleness of the thermoplastic matrices and to lower their melting and glass transition temperatures, thus improving their flexibility and processability [20,21,22]. Plasticization may also influence a variety of other properties, such as density, viscosity, resistance to biological degradation, hardness, resistance to fracture, and degree of crystallinity, among others [22]. Several families of compounds, including polyols (e.g., glycerol [23] and poly(ethylene glycol) (PEG) [24]), triacetin [25], citrate esters (e.g., triethyl citrate (TEC) [26] or acetyl tributyl citrate (ATC) [27]), and epoxidized vegetable oils [19,28], have been investigated as plasticizers in composite formulations. Sugar-based surfactants, e.g., sophorolipids, which are highly desirable given their green character, have also been tested with some pure biobased thermoplastic matrices, such as PLA, PHB and polycaprolactone (PCL) [29], but not in composites of these thermoplastics and cellulose fibers. Alongside plasticizers, coupling agents are also extensively used additives in the composite industry. Such compounds act as a bridge between the matrices and the fibers, thus promoting better interfacial adhesion between them [13]. Maleated coupling agents [30], diisocyanates [31], silanes [32] and even functionalized vegetable oils [33] have been targets of research. Within the catalogue of additives, functionalized vegetable oils are particularly attractive given their sustainability, commercial availability, non-toxicity and relatively low cost [34]. More specifically, epoxidized linseed [19] and soybean [14] oils have been highlighted as additives in green composites of PLA acting simultaneously as coupling agents and plasticizers [14,19,33].

Despite recent studies on the use of additives, more specifically epoxidized oils, on green composites based on PLA or PLA/PHB blends and different plant fibers [28,35,36,37], they have never been used in composites reinforced with micronized cellulose fibers and the examination of crucial properties, such as the melt flow rate (MFR), water uptake, flexural and impact properties, is scarce or non-existent. Moreover, to the best of our knowledge, the effect of surfactants and epoxidized oils as additives in green composites based on PHB has not been evaluated yet. Therefore, in the present study, a sugar-based surfactant and epoxidized linseed oil were individually evaluated as additives in composites with two distinct biobased thermoplastic matrices, namely PLA and PHB, reinforced with micronized bleached eucalyptus kraft pulp (BEKP). The interfacial morphology and the tensile, flexural and impact mechanical performance alongside with the melt flow rate, thermal properties, water uptake capacity and burial behavior in compost medium were thoroughly investigated.

## 2. Materials and Methods

### 2.1. Materials

Poly(hydroxybutyrate) (PHB), grade P226, with a melt flow rate of 10 g·10 min^−1^ (180 °C, 5 kg), density of 1.25 g·cm^−3^ and number-average molecular weight of 22,200 ± 4500 [38], was supplied by Biomer (Schwalbach, Germany). Poly(lactic acid) (PLA), grade 3100HP, with a melt flow rate of 24 g·10 min^−1^ (210 °C, 2.16 kg) and molecular weight of 148 kDa [39] was supplied by NatureWorks (Plymouth, MN, USA). The mechanically treated (micronized) cellulose pulp fibers (average length and width of 332 µm and 12.5 µm, respectively) were obtained through a micronization procedure from eucalyptus bleached kraft pulp and were kindly provided by a Portuguese pulp mill. The epoxidized linseed oil (ELO), composed of stearic (3–5%), palmitic (5–7%), oleic (18–26%), linoleic (14–20%) and linolenic (51–56%) acids, was acquired from Traquisa (Barcelona, Spain). ELO has an oxirane oxygen minimum of 8%, iodine value under 5%, density of 1.1 g·cm^−3^ and viscosity between 800 and 1200 cP. The nonionic surfactant GlucoPure^®^ Sense (GPS), composed of sunflower oil methylglucamide (52%), glycerin (5%), water (10%) and propyleneglycol (33%), was obtained from Clariant (Barcelona, Spain). Compost medium Nutrimais Pulverulento, obtained by selective composting of lignocellulosic residues from forest exploration and from food wastes, was acquired from Nutrimais (Gondomar, Portugal). The specifications of the compost medium are as follows: moisture = 29.23 ± 1.41%, water-holding capacity = 170.33 ± 12.34%, organic matter = 57.66 ± 11.5%, pH = 8.9 ± 0.6, and elemental composition = 32.03 ± 2.66% (total carbon (C)), 2.41 ± 0.48% (total nitrogen (N)), 1.96 ± 0.39 (total potassium (K)), 0.71 ± 0.14% (total magnesium (Mg)), 14.50 ± 2.90% (total calcium (Ca)), and 1.33 ± 0.27% (total phosphorous (P)).

### 2.2. Compounding and Processing of the Composites

Reference composites without additives were obtained by melt mixing the thermoplastic polymers with the micronized cellulose fibers for a fixed fiber load of 40 wt.%. For the formulations with additives, the fibers were pre-mixed with ELO or GPS, followed by the melt mixing with the corresponding matrices. The additives were incorporated in percentages ranging from 2.5 to 7.5 wt.% relative to the total weight of the composites. The formulations were mixed at 180 °C for PLA and 170 °C for PHB, for 15 min at 50 rpm in a Brabender W 30 EHT Plastograph EC mixer (Brabender, Duisburg, Germany) with a total volume capacity of 30 cm^3^. The rectangular and dog-bone shaped test specimens for the characterization assays were produced through injection molding in a Thermo Scientific Haake Minijet II (Thermo Scientific, Waltham, MA, USA). PLA composites were injected at 195 °C with the mold temperature between 100 °C and 125 °C and PHB composites were injected at 185 °C with the mold at 65 °C. The injection pressure was set at 800 bar during 15 s with a post-injection pressure of 200 bar for another 5 s.

### 2.3. Characterization Techniques

The density of the composites was calculated by dividing the test specimens’ weights by their volumes. Rectangular specimens (80 × 10 × 4 mm^3^) with a 3.2 cm^3^ volume were used for density calculations using at least five replicates.

Scanning electron microscopy (SEM) images of the fracture zones of the composites (after the tensile tests) were acquired on a FE-SEM Hitachi SU70 microscope (Hitachi High-Technologies Corporation, Tokyo, Japan) operated at 15.0 kV. Prior to the analysis, the samples were coated with a carbon film.

The determination of the Young’s modulus, tensile strength and elongation at break were performed according to ISO-527-2 (bar type 5A). The tests of at least six dog bone-like specimens with test dimensions of 30 × 4.25 × 2.10 mm^3^ were conducted at a crosshead velocity of 5 mm·min^−1^ using a 10 kN static load cell. The flexural properties of the composites were determined following the three-point loading model according to ISO 178. Five rectangular specimens (80 × 10 × 4 mm^3^) were tested with a 500 N load cell at a velocity of 5 mm·min^−1^. The span length between supports was set at 64 mm. The strain at break was calculated as the ratio between the extension at break and the maximum deflection (20 mm). Both the tensile and flexural tests were conducted on a universal testing machine Instron 5966 (Instron Corporation, Norwood, MA, USA). The unnotched Charpy (edgewise) impact strength of ten specimens with dimensions of 80 × 10 × 4 mm^3^ was acquired on a Ray Ran Universal Pendulum impact system (Ray-Ran Test Equipment Ltd., Nuneaton, UK). The equipment was operating a pendulum of 4 J and the support span was set at 62 mm, according to ISO 179/1eU.

The melt flow rate of the composites was evaluated on a Melt Flow Indexer Devenport (MFR-9) (Ametek, Denmark), according to ASTM D1238. The temperature selected for analysis of the PHB-based composites was 175 °C and for PLA-based composites was 190 °C. At least five cut-offs for each sample were weighted and the melt flow rate (MFR) was calculated as follows:

(1) $$MFR\;\left(g\cdot 10\;\min^{-1}\right)=\frac{600\times m}{t}$$

where *m* is the average mass of the cut-offs, in grams, and *t* is the cut-off time interval, in seconds.

Thermogravimetric analysis (TGA) was carried out on a SETSYS Setaram TGA analyzer (SETARAM Instrumentation, Lyon, France) equipped with a platinum cell. Approximately 10 mg of each sample was heated from room temperature to 800 °C at a rate of 10 °C·min^−1^ under a nitrogen flow.

The differential scanning calorimetry (DSC) thermograms of the matrices and composites were obtained on a Perkin Elmer Diamon DSC unit (PerkinElmer, Waltham, MA, USA). The samples were placed in aluminum capsules and heated from −40 to 220 °C, hold for 5 min at 220 °C to eliminate the thermal history, cool back to −40 °C and reheated from −40 to 220 °C at a heating rate of 10 °C·min^−1^. The results were recorded on the second heating cycle.

To determine the water uptake capacity, three specimens of each sample (60 × 10 × 1 mm^3^) were immersed in distilled water, at room temperature, over a period of 31 days. Then, the specimens were periodically removed from the water, wiped with tissue paper and weighed to determine the weight of the wet specimens. The water uptake (%) at time *t* was calculated as represented in Equation (2):

(2) $$Water\;uptake\;\left(\%\right)=\frac{\left(W_{t}-W_{0}\right)}{W_{0}}\times 100\;$$

where *W*_0_ is the specimen’s initial weight and *W_t_* is the weight of the specimens after immersion for a *t* time period. 

Burial tests of the neat matrices and composites (with and without additives) were performed by measuring the weight loss of the materials after burying them in compost medium. Prior to the tests, composite specimens (60 × 10 × 1 mm^3^) were preconditioned by drying them at 40 °C in an oven for a period of 48 h and the initial mass was recorded (*W*_0_). Then, the specimens were buried in compost in a 1 L container, at a distance of 1 cm from the bottom. Water was added to the containers to adjust the water content to 60 wt.% of the water-holding capacity. The assays were conducted at room temperature (18–25 °C) and the humidity of the compost was constantly adjusted by adding water every couple of days. At the end of 15, 30, 60, 90, 120, 150 and 180 days, three specimens from each sample were retrieved, carefully washed to remove any compost residues and dried at 40 °C for 48 h. Finally, the mass of the specimens was recorded (*W_t_*) to calculate the weight loss according to Equation (3):

(3) $$Weight\;loss\;\left(\%\right)=\frac{W_{0}-W_{t}}{W_{0}}\times 100$$

Statistical analysis of all mechanical properties data was performed using the analysis of variance (ANOVA) and Tukey’s mean comparison test (OriginPro 9.6.5, OriginLab Corporation, MA, USA) with the statistical significance established at *p* < 0.05.

## 3. Results and Discussion

**Figure 1:**
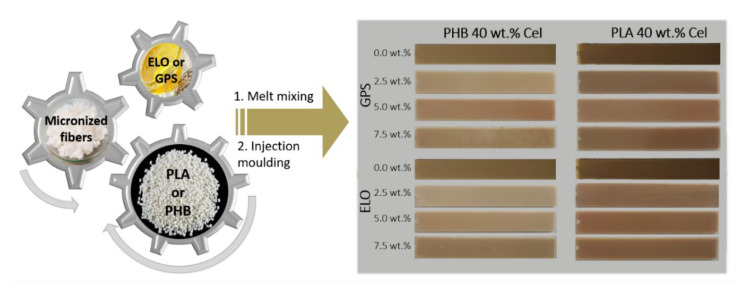
Schematic illustration of the experimental procedure and photographs of the injection-molded specimens of poly(hydroxybutyrate) (PHB) and poly(lactic acid) (PLA)-based composites reinforced with cellulose micronized fibers with different percentages of GPS and ELO additives.

Fully biobased composites composed of PHB or PLA, reinforced with cellulose micronized fibers, were manufactured by melt-mixing and processed by injection molding (Figure 1). Two distinct biobased commercial additives, viz. epoxidized linseed oil (ELO) and a sugar-based surfactant GlucoPure^®^ Sense (GPS), were chosen given their sustainability, non-toxicity, and commercial availability. GPS was selected given that the main constituent is an amphiphilic molecule, potentially enabling interactions with the hydrophilic cellulose and the hydrophobic matrix [40]. Moreover, GPS is also constituted by propylene glycol and glycerin, which are well-known plasticizers. ELO is a recognized plasticizer with acid scavenger abilities, mainly used for poly(vinyl chloride) (PVC), which has also proved efficient in plasticizing green composites and improving the interfacial adhesion between matrices and reinforcements. A fiber load of 40 wt.% was chosen based on the best performance of the PHB and PLA-based composites reported in our previous study [18], as well as by taking into consideration the reinforcement percentage of fibers on commercial petroleum-based composites [41]. The effect of the incorporation of both additives separately, on the properties and performance of the composites, was evaluated.

According to Figure 1, the reference materials, i.e., the composites without additives, are darker than the corresponding materials with either ELO or GPS. This darker color of the reference materials can be attributed to some thermal degradation of cellulose during processing at high temperatures [42]. However, this effect is softened upon the incorporation of ELO and GPS. In fact, Immonen et al. [19] also observed a similar color change on composites made of PLA reinforced with 40 wt.% bleached softwood kraft pulp (BSKP) in the presence of ELO. The lightning was attributed to the plasticizing effect of ELO that reduces the viscosity of the composite material and the friction during processing. From Figure 1, it is also perceptible that composites based on PHB with higher amounts of GPS (7.5 wt.%) have brighter areas on the surface, unlike the ones with 7.5 wt.% ELO. That can be related to an eventual lack of compatibility between the hydrophobic PHB and GPS, which is clearly more hydrophilic than ELO.

The composites, with or without additives, display Fourier Transform Infrared-Attenuated Total Reflection (FTIR-ATR) spectra similar to those of the corresponding thermoplastic polymers (Figure S1), which can be related with the high percentage of the polymers and the relatively low loads of the additives in the composite formulations. Additionally, all the composites have similar density values as listed in Table 1. In fact, and given the relatively low amounts of additives, the average density of the composites is only marginally affected. Even so, a slight decrease trend on the density of the composites can be noted as the percentage of additive increases. That is due to the lower density of the additives (1.06 g·cm^−3^ for both ELO [43] and GPS [44]) in comparison with the thermoplastic matrices (1.25 g·cm^−3^ for PHB [45] and 1.24 g·cm^−3^ for PLA [46]) and cellulose (1.5–1.6 g·cm^−3^) [47]. Thus, the raise in the percentage of a lower density material leads to the slight decrease in the density of the overall composite material.

### 3.1. Morphological Characterization

**Figure 2:**
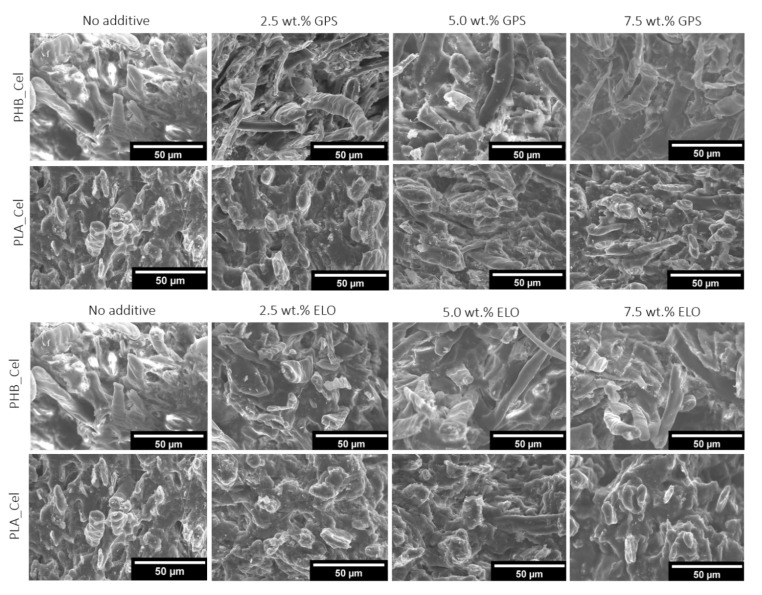
SEM micrographs of the fractured surfaces of PHB- and PLA-based composites without additives and with different contents of GPS and ELO additives.

Scanning electronic microscopy was used to evaluate the morphology of the composites with different additive contents. The SEM micrographs of the fracture zones after tensile testing are displayed in Figure 2. From the analysis of these micrographs, it can be observed that, despite some fiber pull-outs and voids, the compatibility between the fibers and matrices on the composites without additives is relatively good, particularly in the composites based on PLA. This observation is in line with our previous work, where different grades of PLA and PHB matrices were melt compounded with four micronized pulp fibers with distinct aspect ratios [18].

Concerning the composites loaded with the additives, no significant differences were observed when the sugar-based surfactant GPS was added to both PLA and PHB composites, independently of the added content. On the other hand, and regarding the composites with ELO, mainly the ones based on PLA, the interface between fibers and matrix is sometimes indistinguishable, which is indicative of a superior compatibility and interfacial adhesion. That points out that besides acting as a plasticizer, ELO can enhance even further the interaction between the matrix and the fibers. Despite some authors reporting that no major differences could be observed on the SEM micrographs upon incorporation of ELO in green composites of PLA reinforced with birch kraft pulp fibers [33,48], which was attributed to the reasonable interfacial adhesion already observed on the composites without the additive, some other studies revealed similar results to the ones obtained in the present work. For instance, composites of PLA with hazelnut shell flour (HSF) containing 7.5 wt.% ELO showed remarkably different fracture micrographs revealing improved interfacial adhesion [49]. It has been proposed in a few studies that the epoxy groups of the epoxidized oils can react, particularly with cellulose, but also with PLA. More specifically, the ring opening of the epoxy groups by the hydroxyl groups of cellulose, leading to the formation of a covalent ether bond between the epoxidized oil and the cellulosic fibers and the reaction between the epoxy functional groups with the hydroxyl and carboxyl end-groups of PLA, which leads, in the last case, to an ester bond [14,19,49].

### 3.2. Mechanical Properties

#### 3.2.1. Tensile Properties

**Figure 3:**
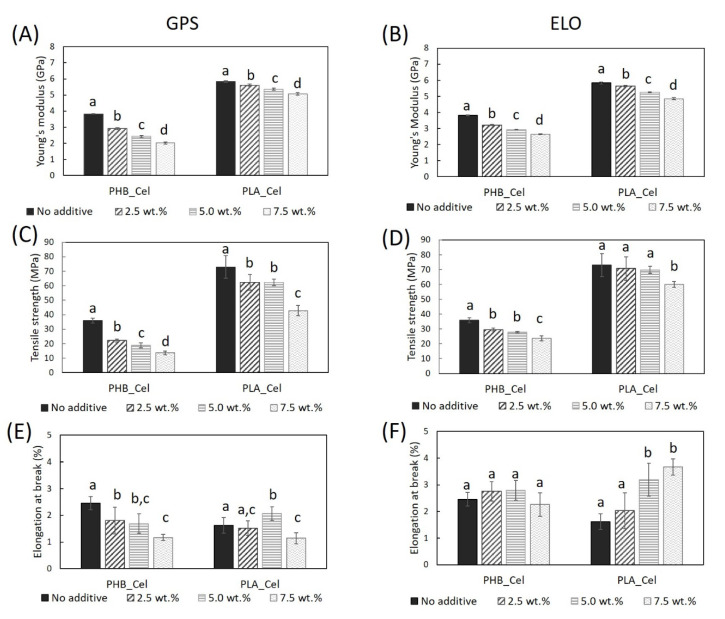
Tensile mechanical properties of the PHB- and PLA-based composites without additives and with GPS (**A**,**C**,**E**) and ELO (**B**,**D**,**F**) additives. Different letters (a,b,c,d) indicate statistically significant differences (*p*  <  0.05).

The tensile performance of the composites with and without additives is presented in Figure 3. The Young’s modulus and tensile strength decreased progressively with the augmentation of the content of the GPS and ELO additives. Moreover, this decline is more pronounced for the PHB-based composites where, for example, the Young’s modulus and tensile strength decreased from 3.83 ± 0.02 GPa and 35.9 ± 1.7 MPa to 2.02 ± 0.06 GPa and 13.6 ± 1.3 MPa, respectively, when 7.5 wt.% of GPS was added. Concerning the elongation at break, while for the PHB-based composites with ELO, the changes were not statistically different, for composites with PLA, this parameter more than doubled (1.62 ± 0.29 to 3.67 ± 0.30%) for higher loads (7.5 wt.%). On the other hand, the GPS caused a decrease in the elongation at break of all the composites, regardless of the thermoplastic matrix. 

It is known that the incorporation of plasticizers, such as PEG or citrate esters, usually results in the decrease in the Young’s modulus and tensile strength, and in the increase in the elongation at break, either for PLA [50] or PHB [51,52] matrices or composites. As for the ELO, literature shows contradictory results for the tensile behavior of PLA-based composites. Taking for instance the example of the HSF-reinforced PLA composites, the addition of ELO led to a decrease in the Young’s modulus and tensile strength and a correspondent increase in the elongation at break [49]. On the contrary, for the PLA composites reinforced with bleached hardwood kraft pulp (BHKP), the addition of ELO increased all three parameters [33]. In another study about PLA composites reinforced with 40 wt.% BSKP, the Young’s modulus and tensile strength increased by 5% and 7%, respectively, for a load of 5 wt.% of ELO (relative to fiber mass). On its turn, the elongation at break was reduced by 12.5%. However, for a higher load, i.e., 12 wt.% of ELO, the composites had inferior Young’s modulus and tensile strength, and higher elongation at break than the reference material, which also corroborates the plasticizing effect of ELO [19]. To the best of our knowledge, ELO was never used in PHB-based composites. However, on a neat matrix of PHB, small amounts of ELO worked as plasticizers, decreasing the tensile strength and modulus, and increasing the elongation at break [53], thus acting in accordance to the present results.

#### 3.2.2. Flexural Properties

**Figure 4:**
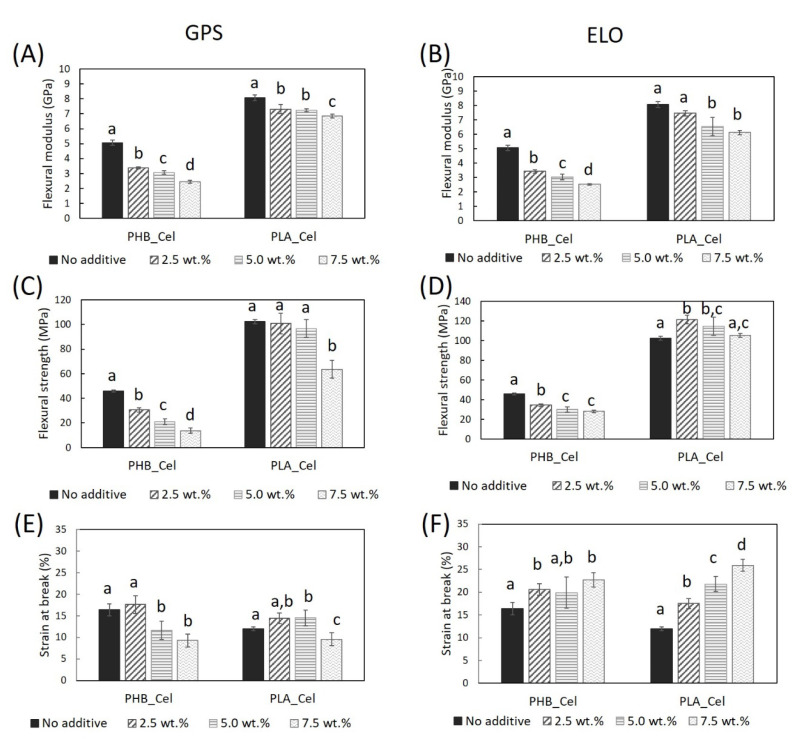
Flexural mechanical properties of the PHB- and PLA-based composites without additives and with GPS (**A**,**C**,**E**) and ELO (**B**,**D**,**F**) additives. Different letters (a,b,c,d) indicate statistically significant differences (*p*  <  0.05).

The flexural properties agree with the tensile data, which means that, for the most part, the incorporation of both additives diminishes the flexural modulus and flexural strength, and that the ELO improves the flexural strain at break of the composites (Figure 4). The flexural modulus of the PHB-based composites containing 7.5 wt.% of additives decreased to less than half of the initial value (from 5.1 ± 0.2 GPa to 2.4 ± 0.1 for GPS and 2.5 ± 0.1 GPa for ELO), while the reduction for the PLA-based composites is much less noticeable. For instance, there are no statistical differences between the flexural modulus of PLA_Cel without ELO and with 2.5 wt.% ELO and between the PLA_Cel with 5.0 wt.% or 7.5 wt.%. Concerning the strain at break, with the incorporation of 7.5 wt.% of ELO, a raise of 39% and 115% was observed for PHB and PLA-based composites, respectively. This increase in the flexibility of the composites is also due to the plasticizing effect of ELO, which decreases the intermolecular forces between the polymeric chains, thus reducing the brittleness of the materials, consequently increasing their flexibility, ductility and extensibility [49,54]. Similar trends on the flexural behavior have been reported by Balart et al. [49] for composites of PLA reinforced with HSF and using ELO as plasticizer. On the other hand, the flexibility of the composites with GPS, represented by the strain at break, decreased with the incorporation of the sugar-based surfactant. This behavior is certainly related to the lack of compatibility of this hydrophilic additive with the thermoplastic matrices, as previously observed on the SEM images, particularly for composites of PHB.

#### 3.2.3. Impact Properties

**Figure 5:**
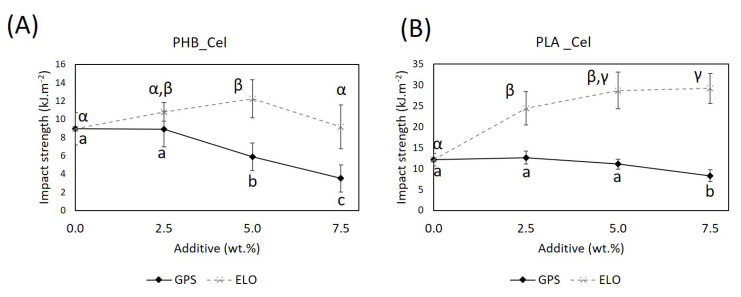
Impact strength of the PHB- (**A**) and PLA-based composites (**B**) without additives and with different loads of GPS and ELO additives (the lines are for visual guidance only). Different letters (a,b,c) or (α,β,γ) indicate statistically significant differences (*p*  <  0.05).

The impact resistance is one of the mechanical parameters that is often seriously compromised in green composites, either due to the brittleness of the matrices or its poor interfacial adhesion with the cellulosic fibers. Therefore, the understanding of the effect of both additives on the Charpy impact strength of the composites is of utmost importance (Figure 5). As observed from the results, the incorporation of ELO, at a 5 wt.% load, raises the impact strength of the composites by 36.7% and 135.7% for PHB and PLA-based composites, respectively. These outstanding improvements, especially in composites with PLA, are the result of the plasticizing effect of ELO combined with the enhanced interfacial adhesion induced by this additive. Both the plasticizer effect of ELO, which reduces the brittleness of ternary mixtures, and the enhanced interfacial adhesion between the polymeric matrices and the reinforcing fibers contribute to averting the initiation and propagation of cracks [54]. A plateau on the impact resistance of the materials is achieved for 5 wt.% of ELO. A further increase in the additive content does not cause any significant upgrade on the impact resistance, most likely because after that threshold, the ELO molecules start to interact more with themselves than with cellulosic fibers or even with the PHB or PLA [55]. It is worth mentioning that the use of ELO in the present work brings the values of the impact strength of these green composites close to those of biocomposites based on PP and PE currently available on the market (33 to 42 kJ·m^−2^) [41,56]. 

Regarding the influence of the sugar-based surfactant GPS, this additive did not contribute to the improvement of the impact strength of the composite materials. Instead, the impact strength decreased for higher loads of the additive, mainly for PHB-based composites. The lack of compatibility between the hydrophilic constituents of GPS and the hydrophobic matrices, previously mentioned, are probably the main cause for the loss of the resistance to impact. In fact, these results are also in agreement with the outcomes of the tensile and flexural tests previously described.

Several other works have previously reported improvements on the impact properties of green composites with PLA using various epoxidized oils, such as epoxidized soybean (ESO) [55], linseed (ELO) [19], palm (EPO) [57] and jatropha (EJO) oils [36]. Yet, the improvements were modest in comparison with the results obtained in the present study. For instance, an ELO load of 8 wt.% relative to the weight of the BSKP fibers yielded a 37% increase in the impact resistance, raising the Charpy unnotched impact strength to only 18 kJ·m^−2^ [19]. In a similar attempt to induce better mechanical properties, the utilization of ELO in composites with BHKP increased the impact strength by 25.9% [33]. The use of low aspect ratio-micronized fibers in the present study certainly contributed to the better performance of the materials when compared with the aforementioned studies. As disclosed in a previous work, the use of fibers with smaller aspect ratios reduces the formation of defects and consequent initiation and propagation of cracks [18].

Concerning the PHB-based composite materials, and as far as our literature search could verify, no data are available regarding the use of any sugar-based surfactants or epoxidized oils as additives. However, on a neat PHB matrix, ELO proved useful for increasing the impact strength [53]. Moreover, plasticizers, such as soybean oil (SO), ESO or TEC, have been tested in films made of a PHB copolymer, more specifically poly(3-hydroxybutyrate-*co*–3-hydroxyvalerate) (PHBV). Both ESO and TEC plasticizers promoted considerable improvements on the impact strength of the blends [58].

### 3.3. Melt Flow Rate

**Figure 6:**
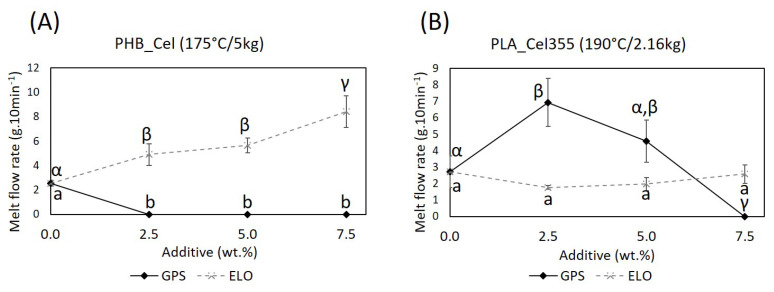
Melt flow rate of the PHB- (**A**) and PLA-based composites (**B**), without additives and with different loads of GPS and ELO additives (the lines are for visual guidance only). Different letters (a,b) or (α,β,γ) indicate statistically significant differences (*p*  <  0.05).

The melt flow rate, expressed in grams of composite extruded through a nozzle in ten minutes, and measured at a constant temperature when a standard weight is applied, is an indirect measurement of melt viscosity and molecular weight of the materials and an indication of their processability [59,60]. The variation of the MFR of composites as a function of the load of additives is illustrated in Figure 6. With the additive GPS, none of the composites based on PHB flowed at all, at any of the percentages. The lack of flowability of these composites is certainly related with the migration of the additive. In fact, the clear separation of a liquid with the same color and consistency of GPS observed during the MFR assays provides evidence of phase separation. As a matter of fact, the diffusion and leaching of the plasticizer from the bulk material to the surroundings is one of the main disadvantages reported for the use of this class of additives, which can seriously compromise the performance of the plasticized composite materials [61]. Several characteristics of the plasticizer, such as the type, molecular weight, branching degree and polarity, can affect its migration [20,22]. The compatibility of the plasticizer with the thermoplastic polymer and some conditions, such as the temperature, can also have an effect on this behavior [20,22]. Thus, considering that low molecular weight plasticizers tend to have higher migration rates and the high temperatures also favor the migration [62], it is not surprising that GPS, given its formulation and the temperature at which the MFR assay is conducted, is highly prone to migration. Moreover, the lack of compatibility between GPS and PHB, as noticed before, certainly contributes to its diffusion from the bulk material to the surroundings. As for the PLA-based composites, at low percentages of GPS (2.5 wt.%), the migration phenomenon did not take place and a raise on the MFR from 2.71 to 6.93 g·10 min^−1^ could be observed. With increasing amounts of this additive (higher than 5.0 wt.%), the melt flow rate started to drop and the separation of the additive from the composite also became clear.

On the contrary, ELO has a low migration tendency [63]. The melt flow rate of PHB-based composites with 7.5 wt.% ELO experiences a remarkable increase of 230%, from 2.55 to 8.42 g·10 min^−1^. Such improvement is undoubtedly related to the plasticizing effect of ELO in PHB based composites, which reflects the inferences made from the mechanical assays. Aside from the increase in the molecular mobility of the polyester chains, the plasticizer reduces the fiber/fiber and fiber/matrix friction, which contributes to the improvement of the melt flowability [24]. For the composites with PLA, the addition of ELO did not change the MFR of the composites. As depicted in the SEM micrographs (Figure 2), ELO promotes the increase in the interfacial adhesion of the fibers with PLA, which prevents the melt flow rate from increasing. Immonen et al. [19] also claimed that a decrease was expected in the apparent viscosity of the PLA/BSKP composites with ELO, if the epoxidized oil was working solely as a plasticizer. However, due to the improved compatibilization of the fibers with PLA caused by the addition of ELO, an increase was noted instead. 

Although of extreme importance, the influence of the addition of eco-friendly plasticizers in the melt flow rate of green composites has not been the subject of intensive research. Previous studies reported that composites of bamboo reinforced PLA plasticized with PEG had better flowability than the corresponding composites without additives [24] and that ESO could be used as plasticizer for neat PLA [64]. However, to the best of our knowledge, the melt flow rate of green composites with epoxidized oils or sugar-based surfactants as additives was not yet reported. The present work demonstrates that ELO can improve the processability of PHB-based composites, and GPS, at low amounts, can also contribute to a better flowability of PLA-based composite materials.

### 3.4. Thermal Properties

Differential scanning calorimetry was performed to evaluate the impact of the additives on the onset and peak melting temperature (T_m_) of the composites. The results portrayed in Figure S2 and summarized in Table 2 show that, unlike PLA-based composites where only one melting peak can be observed between 160.4–169.3 °C, the PHB ones have two melting peaks (135.6–149.2 °C and 152.1–163.0 °C). The existence of double or multiple peaks is not uncommon for PHB and has also been reported elsewhere [38]. That behavior can be related with the partial melting and recrystallization and remelting (mrr) process, to the melting of crystals with different lamellar thicknesses or to the melting of different crystalline structures [65].

The second heating scans also reveal that, for the PHB-based composites, the incorporation of increasing amounts of GPS gradually lower both the onset and peak melting temperatures of the composites. For instance, the addition of 7.5 wt.% of GPS leads the melting temperatures’ peak to drop by 12.6 °C and 10.3 °C. On the other hand, in composites with PLA, the peak melting temperature is increased for lower loads of GPS and decreased 4.6 °C for 7.5 wt.%. With ELO, a reduction on the melting temperature was only observed for composites with PHB, mainly at higher concentrations. All the aforementioned decreases are related to the plasticizing action of the additives, which reduces the intermolecular interactions, consequently lowering the melting point [27]. Similar outcomes on the lowering of the melting point as a cause of plasticization have also been reported, for example, for PHB [53] and PLA [66] matrices with epoxidized vegetable oils and also in green composites of PHB plasticized with ATC [27]. For the case of the composites with PLA and ELO, the melting point shifts to higher temperatures with the incorporation of this additive. For instance, 5 wt.% ELO raises the melting point from 165.0 °C to 168.9 °C. It is suggested that the increased interaction between the components of the ternary mixture with the incorporation of ELO is responsible for the increase in the melting peak. These results can once more be linked to the enhanced interfacial morphology discussed above. Surprisingly, in previous works about the addition of epoxidized oils in green composites of PLA, the authors reported lower melting temperatures on the composites with the epoxidized oil, which was mainly associated to its plasticizing effect [14,19,33].

The thermal stability of the composites, accessed through TGA, is also presented in Table 2 and Figure S3. The observation of two degradation steps in the composites with PHB and only one in the composites with PLA was already reported in a previous work [18]. For composites with PHB, the first step, which corresponds to the degradation of the thermoplastic polymer, shifts to lower temperatures when the GPS load is over 5.0 wt.%. Additionally, for the second step, which is related with the degradation of the cellulosic fibers, the incorporation of GPS promotes a decrease in the maximum degradation temperature from 351.2 °C to 336.5 °C. The low compatibility of GPS with the cellulosic fibers and with PHB, as seen on the test specimens on Figure 1, may play an important role on the decrease in the thermal stability of the PHB-based composites with GPS. For the PLA-based composite materials, the maximum degradation temperature also decreased from 336.2 °C to 332.9 °C with the addition of 7.5 wt.% GPS. With regards to ELO, for composites with PHB, a 5 wt.% load gives the best thermal stability with maximum degradation temperatures of 283.0 °C and 352.5 °C. For PLA-based materials, the maximum degradation temperature is increased up to 5.5 °C. This increase in the thermal stability has been attributed in other works to the improved interaction between the ELO with the polymer chains, leading to the formation of a barrier on the surface which restricts the permeability of volatile compounds to the exterior [49,53]. Similar to the action of ELO in the present study, the incorporation of other epoxidized vegetable oils, such as EPO [54] and epoxidized palm and soybean oil (ESPO) [67], have also shown improved thermal stability, particularly with PLA.

### 3.5. Water Uptake Capacity

**Figure 7:**
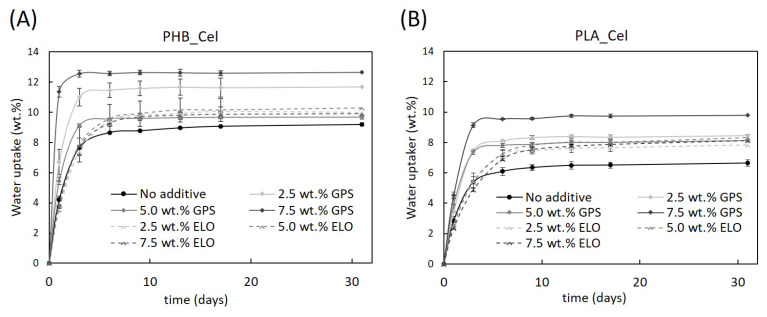
Water uptake capacity of the PHB- (**A**) and PLA-based composites (**B**) without additives and with different loads of GPS and ELO additives (the lines are for visual guidance only).

It is well known that hydrophobic matrices have almost negligible water uptakes. For instance, the maximum water absorption of the neat PHB and PLA used in the present work is under 1.3 ± 0.1%, after 30 days of immersion [18]. On the other hand, cellulose is hydrophilic and, when added for the manufacturing of composite materials, may cause some level of swelling and dimensional instability [68,69]. Moreover, the stress generated by the swelling of the fibers may lead to the occurrence of cracks, seriously compromising the mechanical performance of the composites [70]. In Figure 7, it is perceptible that composites with 40 wt.% of cellulose fibers absorbed water quickly in an early stage, reaching a plateau at the end of approximately 5 days. With the addition of the additives, the water uptake of the composites increased, for both PHB- and PLA-based materials. For example, the water uptake raised from 9.7 % (no additive) to a maximum of 12.6% (GPS) and 9.9% (ELO), for composites based on PHB, and from 6.6 % (no additive) to 9.8 % (GPS) and 8.1% (ELO), for composites based on PLA. Such increments in water uptake, explicitly more expressive for GPS, must be related to its plasticizing effect and hydrophilic character: the plasticizer molecules weaken the intermolecular interactions between the polymeric chains, increasing the ductility of the matrices, ultimately allowing fibers to absorb more water and to swell more easily. The swollen fibers may lead to the formation of cracks, which further contributes to the increase in the water uptake [71].

Also visible on the results presented in Figure 7 is that, while for composites with GPS, higher percentages of the additive resulted in higher amounts of water absorbed by the composite, the water uptake capacity remained similar for composites with increasing amounts of ELO. That can be explained by taking into consideration that GPS has in its composition considerable amounts of hydrophilic molecules (glycerin and propylene glycol), which certainly contribute to higher water absorption. Furthermore, the sugar portion of the surfactant may also contribute to this outcome. Thus, as the amount of GPS is increased in the composites, and with it the content of hydrophilic moieties, the affinity for water gradually increases. On the contrary, ELO is a more hydrophobic molecule which, in addition, and as mentioned before, seems to improve the interfacial adhesion, thus reducing the number of gaps in the interfacial region [49,70]. In fact, Balart et al. [71] investigated the water uptake of PLA composites with HSF in the presence of ELO, and also concluded that ELO contributed to the increase in the water uptake and that the load of this additive was not a differentiating factor [71].

### 3.6. Degradation in Compost Medium

**Figure 8:**
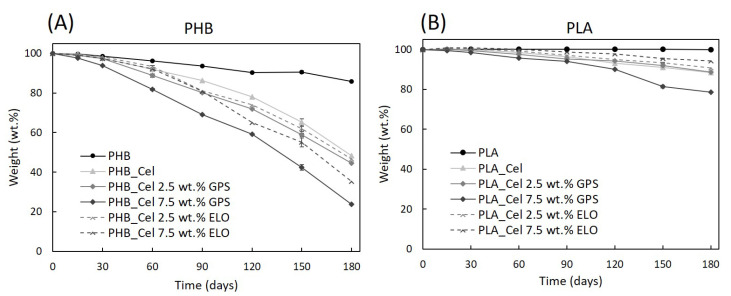
Weight loss as a function of time for the PHB- (**A**) and PLA-based composites (**B**) without additives and with different loads of GPS and ELO additives. The standard deviations (not shown) are under 7.6% for PHB-based materials and under 3.9% for PLA-based materials, and the lines are for visual guidance only.

The evaluation of the degradation of the composite materials in environmental conditions is becoming increasingly important. In reality, the alleged biodegradability of PLA and PHB is one of the main motivations behind their use in green composites with natural fibers and one of the leading advantages over traditional PP- or PE-based composites [6]. Since several conditions, such as the temperature and moisture of the soil/compost medium, the characteristics of the polymers, namely chemical composition, crystallinity, molecular weight and the presence of additives, have an influence on the biodegradation of the composites [72], it is also key to test the effect of the additives used in this work on the biodegradation profile of the obtained composites. Moreover, and considering that additives influence the properties of the composites, it is also expected that they interfere with the resistance to biological degradation [20]. Thus, the weight loss of the composites with the additives and the reference materials was evaluated under controlled conditions, in compost medium, and the visual representation of the data is illustrated in Figure 8.

First, it is evident that the neat matrices have different degradation rates. While the pristine PHB lost 14.2% of its initial weight, the mass of pure PLA specimens was virtually unchanged over six months. That can be explained by taking into consideration that PHB and PLA have different biodegradation mechanisms [10,73]. If on the one hand, PHB biodegradation is solely enzymatic, on the other hand, the biodegradation of PLA first starts with non-enzymatic hydrolysis, followed by enzymatic degradation [10,73]. The non-enzymatic hydrolysis begins with water absorption that allows for a random cleavage of the ester bonds of the polymer, resulting in lower molecular weight oligomers and lactic acid. Such hydrolysis occurs preferably at temperatures above the PLA glass transition temperature (55–62 °C) [10,74]. Given that the present compost burial experiments were conducted at room temperature (18–25 °C), that explains why PLA did not show any signs of degradation. Similar results were obtained by Zhang et al. [75] who also demonstrated that PHB could in fact be degraded in compost, while PLA did not show any weight loss at room temperature. In yet another study, in a one-year test run, injection molded tensile bars of PLA did not show any signs of degradation at 25 °C both on compost and soil. However, at temperatures near thermophilic conditions (50 °C), weight losses of near 45% were recorded after only 4 weeks [76].

**Figure 9:**
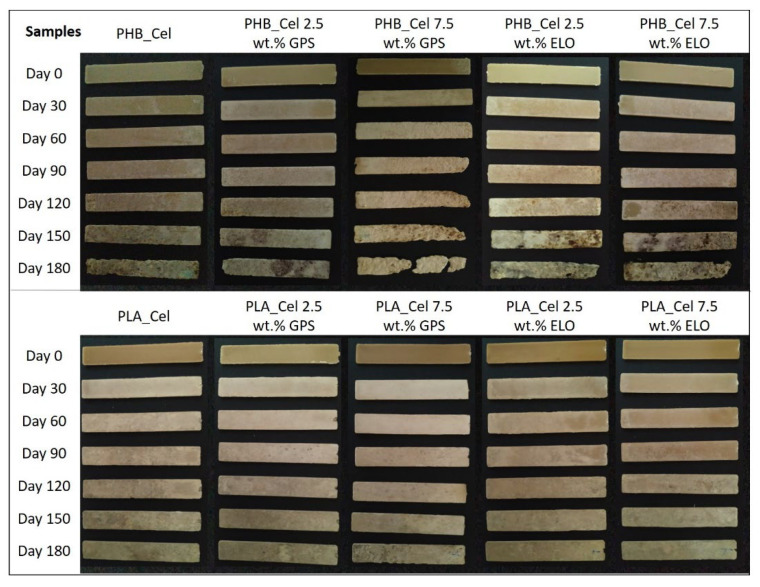
Digital photographs of the test specimens prior and after burial tests in compost medium for 30, 60, 90, 12 and 180 days.

As cellulosic fibers are easily degraded under environmental conditions [77], their incorporation into the biobased matrices promoted a considerable increase in the weight loss of the composites. A closer look at the test specimens in Figure 9 and the degradation profiles displayed in Figure 8 reveals that the composites with PHB have higher weight losses than the ones with PLA, which is obviously related to the degradation of the matrices, or in the case of PLA, of its absence. As PLA showed no degradation at these specific conditions, the weight loss of the composites is only attributed to the degradation of cellulose fibers, while for PHB-based composites, the weight loss is due to the combination of the degradation of cellulose and PHB. It can also be observed that the degradation rate speeds up with time, which is a consequence of surface erosion that facilitates the diffusion of water and extracellular enzymes from the microorganisms, making the polymers more easily accessible for degradation [72].

With the exception of PLA-based composites with ELO, the composites with additives showed higher weight losses than the reference materials without additives. For instance, the incorporation of 7.5 wt.% of GPS increases the weight loss from 53.4% to 76.2% in composites with PHB and from 11.6% to 21.4% in composites with PLA. Such results can be correlated with the water uptake capacity (Figure 7), which, as discussed before, continuously raised with the load of GPS. Higher water absorption promotes the swelling of the fibers and increases the availability of bulk material to enzymatic activity, thus resulting in higher degradation rates [77]. Another possible contributing factor is the leaching of the hydrophilic components of GPS, leading to an increase in internal surface area. A similar conclusion on the leaching of hydrophilic plasticizers and its effect on the biodegradation was disclosed on PHB films plasticized with triethyl citrate [78]. For the case of the composites with ELO, this additive still raises the weight loss in the mixtures with PHB, mainly because of its plasticizing effect and consequent increase in the water uptake. However, although the water uptake is still higher in PLA composites with ELO than in the reference materials, the weight loss is inferior in the compost burial experiments, which can be associated with the improved interfacial adhesion between fibers and PLA promoted by ELO, as previously discussed. Since the biodegradation of composites mainly begins around the interface between the matrix and the reinforcements, an improvement on the interfacial adhesion decelerates the hydrolysis mechanism of the composites [77,79]. Other authors reported a similar outcome with the use of ELO on PLA-based composites. Despite the slight increase in the water uptake of the material with the incorporation of ELO, the disintegration rate in composting conditions was found to be lower, which was credited to the coupling effect of ELO [49,71].

## 4. Conclusions

Two eco-friendly and commercially available additives, namely an epoxidized linseed oil and the sugar-based surfactant GlucoPure^®^ Sense, were successfully incorporated on green composites of PLA and PHB reinforced with cellulose micronized fibers. GPS, a mixture of a sugar-based surfactant with propylene glycol and glycerin, mostly functioned as a plasticizer, making the composite materials more ductile, and consequently decreasing their tensile and flexural properties. This additive improved the composite degradation rates on compost medium and, in certain amounts, was beneficial to improve the melt flow rate in composites with PLA. Nevertheless, this additive is not suitable for PHB-based materials since it lacks compatibility with this thermoplastic polymer, thus compromising its processability. Regarding ELO, it performed differently on PLA and PHB-based composites. If from one side, on the composites with PHB, ELO mainly caused a drastic improvement on the melt flow rate, on the other side, in composites with PLA, it also enhanced the interfacial adhesion, which contributed to an outstanding increase in the impact resistance of the green composites. Both additives led to an increase in the water uptake and, for most cases, also accelerated the weight loss on compost under environmental conditions.

Overall, ELO and GPS additives proved to be viable as functional additives to improve the performance and processability of green composites. Properties, such as the impact strength and melt flow rate, may be tuned using different amounts of such additives. Moreover, their use is of simple implementation on industrial procedures with the additional advantage of being entirely environmentally friendly.

## Figures and Tables

**Table 1:** Density of the PHB- and PLA-based composites reinforced with cellulose micronized fibers without additives and with different percentages of GPS and ELO additives.

Sample	Density (g·cm^−3^)	Sample	Density (g·cm^−3^)
PHB_Cel	1.34 ± 0.01	PLA_Cel	1.38 ± 0.00
PHB_Cel 2.5 wt.% GPS	1.34 ± 0.02	PLA_Cel 2.5 wt.% GPS	1.36 ± 0.02
PHB_Cel 5.0 wt.% GPS	1.33 ± 0.02	PLA_Cel 5.0 wt.% GPS	1.37 ± 0.00
PHB_Cel 7.5 wt.% GPS	1.30 ± 0.03	PLA_Cel 7.5 wt.% GPS	1.37 ± 0.01
PHB_Cel 2.5 wt.% ELO	1.34 ± 0.01	PLA_Cel 2.5 wt.% ELO	1.37 ± 0.00
PHB_Cel 5.0 wt.% ELO	1.33 ± 0.02	PLA_Cel 5.0 wt.% ELO	1.36 ± 0.00
PHB_Cel 7.5 wt.% ELO	1.30 ± 0.02	PLA_Cel 7.5 wt.% ELO	1.35 ± 0.00

**Table 2:** DSC and TGA data of the PHB- and PLA-based composites without additives and with different loads of GPS and ELO additives.

Samples	DSC				TGA	
	Onset_1_ (°C)	Tm_1_ (°C)	Onset_2_ (°C)	Tm_2_ (°C)	T_max1_ (°C)	T_max2_ (°C)
PHB_Cel	142.7	148.2	154.2	162.4	276.2	351.2
PHB_Cel 2.5 wt.% GPS	142.1	147.5	153.5	162.2	281.4	338.1
PHB_Cel 5.0 wt.% GPS	136.9	141.9	149.0	157.2	275.4	335.9
PHB_Cel 7.5 wt.% GPS	126.3	135.6	141.7	152.1	269.2	336.5
PHB_Cel 2.5 wt.% ELO	144.0	149.2	155.9	163.0	283.1	346.9
PHB_Cel 5.0 wt.% ELO	141.4	146.8	153.1	160.9	283.0	352.5
PHB_Cel 7.5 wt.% ELO	140.6	146.0	152.6	160.9	276.4	349.7
PLA_Cel	155.7	165.0	-	-	336.2	-
PLA_Cel 2.5 wt.% GPS	159.0	166.8	-	-	338.3	-
PLA_Cel 5.0 wt.% GPS	150.1	168.1	-	-	331.8	-
PLA_Cel 7.5 wt.% GPS	150.7	160.4	-	-	332.9	-
PLA_Cel 2.5 wt.% ELO	160.3	169.3	-	-	341.7	-
PLA_Cel 5.0 wt.% ELO	158.9	168.9	-	-	339.4	-
PLA_Cel 7.5 wt.% ELO	152.2	166.9	-	-	341.5	-

## Supplementary Materials

The following supporting information can be downloaded at: https://www.mdpi.com/article/10.3390/polym14173451/s1, Figure S1: FTIR-ATR spectra of PHB, PHB_Cel and PHB_Cel 7.5 wt.% ELO (A) and PLA, PLA_Cel and PLA_Cel 7.5 wt.% ELO (B), Figure S2: Differential scanning calorimetry curves of PHB- (A,C) and PLA-based composites (B,D) without and with different loads of GPS and ELO additives, Figure S3: Thermogravimetric and derivative curves of PHB- (A) PLA-based composites (B) without and with different loads of GPS and ELO additives.
