# Supplementary material for: Improving the Processability and Performance of Micronized Fiber-Reinforced Green Composites through the Use of Biobased Additives
Source: Polymers (Basel). 2022 Aug 24;14(17):3451. doi: 10.3390/polym14173451 (PMC9459967; doi:10.3390/polym14173451)
Supplement: Supplementary file 1 [file polymers-14-03451-s001.zip › polymers-1832330-supplementary.pdf]

polymers
MDPI

Supplementary Files

Improving the Processability and Performance of Micronized Fiber-Reinforced Green Composites through the Use of Biobased Additives

Bruno F. A. Valente [1], Armando J. D. Silvestre [1], Carlos Pascoal Neto [2], Carla Vilela [1] and Carmen S. R. Freire [1,*]

[1] CICECO–Aveiro Institute of Materials, Department of Chemistry, University of Aveiro, 3810-193 Aveiro, Portugal
[2] RAIZ, Research Institute of Forest and Paper (The Navigator Company), Rua José Estevão, Eixo, 3800-783 Aveiro, Portugal
* Correspondence: cfreire@ua.pt

Citation: Valente, B.F.A.; Silvestre, A.J.D.; Neto, C.P.; Vilela, C.; Freire, C.S.R. Improving the Processability and Performance of Micronized Fiber-Reinforced Green Composites through the Use of Biobased Additives. *Polymers* **2022**, *14*, 3451. https://doi.org/10.3390/polym14173451

Academic Editor: Evgenia G. Korzhikova-Vlakh

Received: 08 July 2022
Accepted: 12 August 2022
Published: 24 August 2022

Publisher's Note: MDPI stays neutral with regard to jurisdictional claims in published maps and institutional affiliations.

Copyright: © 2022 by the authors. Licensee MDPI, Basel, Switzerland. This article is an open access article distributed under the terms and conditions of the Creative Commons Attribution (CC BY) license (https://creativecommons.org/licenses/by/4.0/).

Fourier Transform Infrared-Attenuated Total Reflection

Fourier Transform Infrared-Attenuated Total Reflection (FTIR–ATR) spectra were obtained on a Perkin-Elmer FT–IR System Spectrum BX spectrophotometer (Perkin-Elmer Inc., Waltham, MA, USA) equipped with a single horizontal Golden Gate ATR cell, over the range of 600–4000 cm^{-1} at a resolution of 4 cm^{-1} over 32 scans.

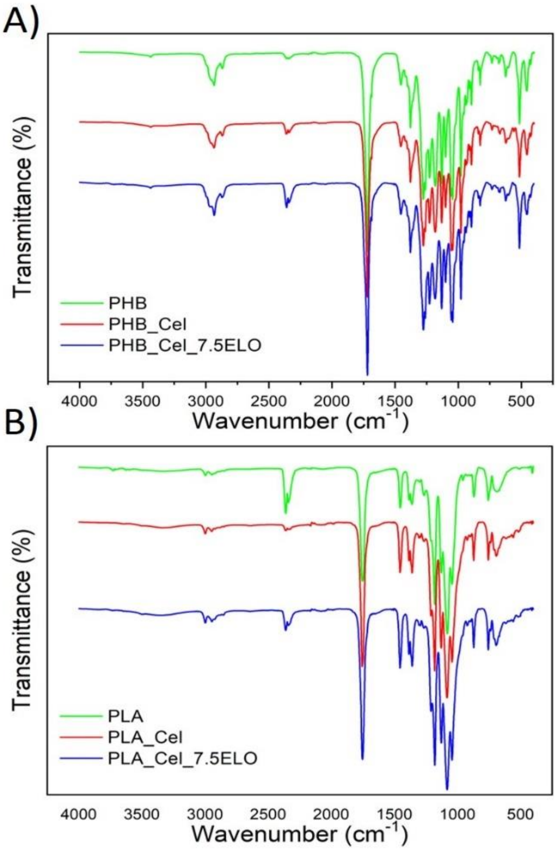

Figure S1. FTIR-ATR spectra of PHB, PHB_Cel and PHB_Cel 7.5 wt.% ELO (**A**) and PLA, PLA_Cel and PLA_Cel 7.5 wt.% ELO (**B**).

Differential Scanning Calorimetry

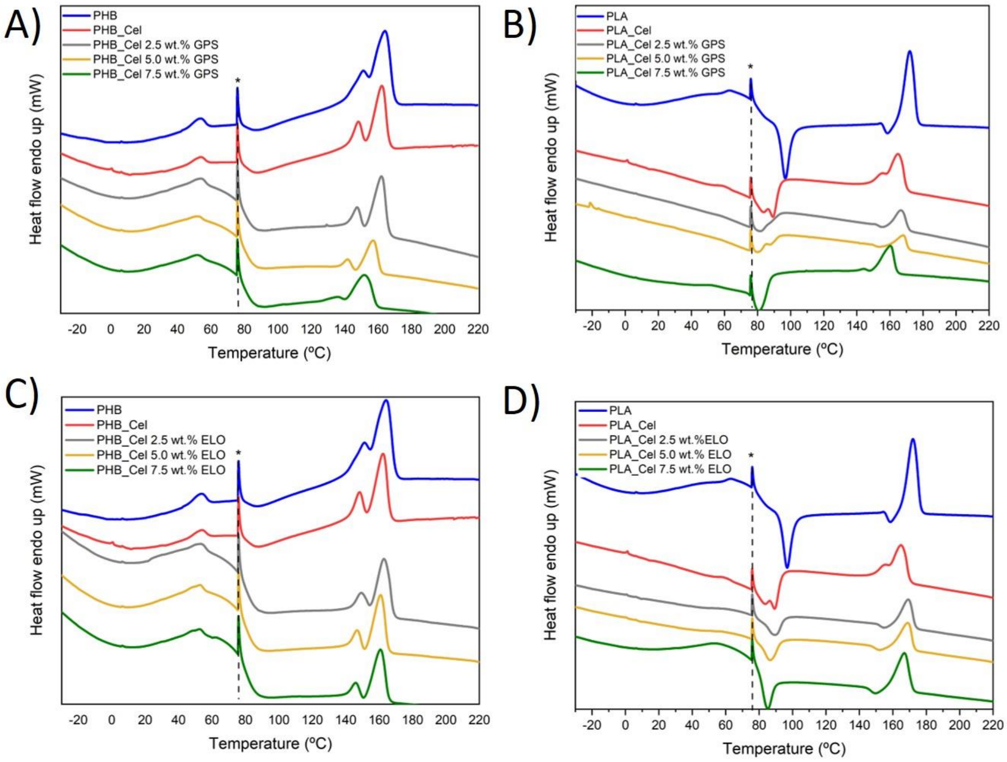

Figure S2. Differenial scanning calorimetry curves of PHB- (**A**,**C**) and PLA-based composites (**B**,**D**) without and with different loads of GPS and ELO additives. * The peaks at 76 °C present in all samples are due to an equipment artefact.

Thermogravimetric Analysis

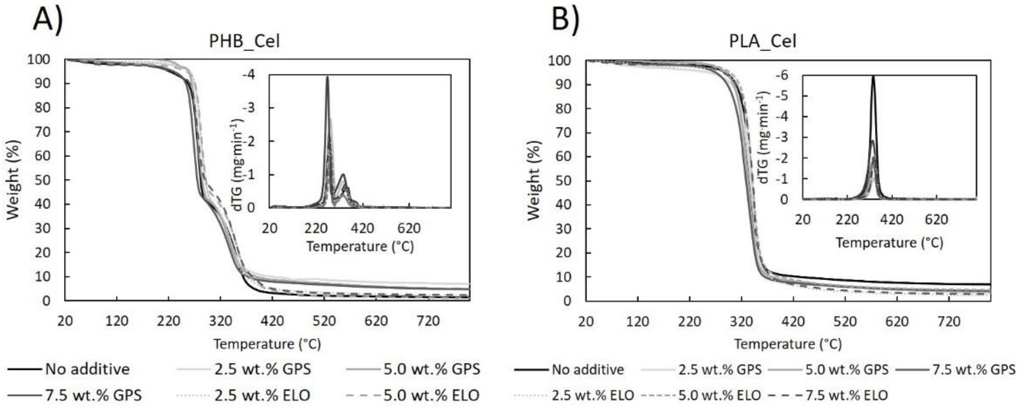

Figure S3. Thermogravimetric and derivative curves of PHB- (**A**) PLA-based composites (**B**) without and with different loads of GPS and ELO additives.
